# Genome-Wide Identification and Characterization of PRR Gene Family and their Diurnal Rhythmic Expression Profile in Maize

**DOI:** 10.1155/2022/6941607

**Published:** 2022-05-16

**Authors:** Cuiling Wang, Leili Wang, Qingqing Liu, Yanling Zhang, Keqing Dong

**Affiliations:** Department of Agronomy, Henan University of Science and Technology, Luoyang 471023, China

## Abstract

As essential components of the circadian clock, the pseudo-response regulator (PRR) gene family plays critical roles in plant photoperiod pathway. In this study, we performed a genome-wide identification and a systematic analysis of the PRR gene family in maize. Nine *ZmPRR*s were identified, and the gene structure, conserved motif, evolution relationship, and expression pattern of *ZmPRRs* were analyzed comprehensively. Phylogenetic analysis indicated that the nine *ZmPRR* genes were divided into three groups, except for *ZmPRR73*, two of which were highly homologous to each of the *AtPRR* or *OsPRR* quintet members. Promoter cis-element analysis of *ZmPRRs* demonstrated that they might be involved in multiple signaling transduction pathways, such as light response, biological or abiotic stress response, and hormone response. qRT-PCR analysis revealed that the levels of *ZmPRRs* transcripts varied considerably and exhibited a diurnal rhythmic oscillation expression pattern in the given 24-h period under both SD and LD conditions, which indicated that the level of transcription of *ZmPRRs* expression is subjected to a circadian rhythm and modulated by light and the circadian clock. The present study will provide an insight into further exploring the biological function and regulatory mechanism of *ZmPRR* genes in circadian rhythm and response to photoperiod in maize.

## 1. Introduction

The perception of daily changes in photoperiod and temperature enables plants to adapt to different latitudes and achieve successful reproduction. The circadian clock is an autonomous oscillator that produces endogenous biological rhythms of a period of about 24 h and plays crucial roles in coordinating the development and metabolism with daily and seasonal changes through the synchronous expression of genes involved in related biological processes. Many clock-associated genes constitute an interlocked transcription and translation feedback loops to maintain the function of the circadian clock, in which clock-associated genes regulate each other to generate oscillations with expression peaks at specific times during the daily cycle [1–5]. Pseudo-response regulators are important components of the core of circadian clock. The *Arabidopsis* PRR family has five members: TIMING OF CAB EXPRESSION 1(TOC1/PRR1), PRR3, PRR5, PRR7, and PRR9.

Numerous studies have indicated the significant roles of PRRs in circadian clock [6]. TOC1 is an essential component of the *Arabidopsis* circadian oscillator, which exerts its function by directly repressing the transcription of morning-expressed oscillator genes CIRCADIAN CLOCK-ASSOCIATED 1 (CCA1) and LATE ELONGATED HYPOCOTYL (LHY) at the core of the clock [7, 8]. PRR9, PRR7, and *PRR5* are transcription repressors and repress the transcription of *CCA1*, *LHY*, *REVEILLE 8* (*RVE8*), *NIGHT LIGHT-INDUCIBLE AND CLOCK-REGULATED 1* (*LNK1*), *LNK2*, *LNK3*, *LNK4*, and *PRR* genes expressed during earlier phases [4]. In addition, PRRs were reported to directly interact with and stabilize CONSTANS (CO) protein, thereby increasing the binding of CO to the promoter of FLOWERING LOCUS T (FT), leading to enhanced *FT* transcription and early flowering under long-day conditions [9].

The PRR gene family has a highly conserved protein structure. Each member contains a pseudo-receiver (PR) domain of approximately 120 amino acids at its N-terminus and a CCT motif of approximately 50 amino acids at the C-terminal end [10–12]. The PR domain of PRRs allows oligomerization between the PRRs and bridges interactions of the PRRs and other proteins [5, 7, 11, 13–15]. The transcriptional repression activity of PRRs relies on the presence of a functional CCT domain to recognize the target genes [7, 16–18]. PRR9, PRR7, and PRR5 have a transcriptional repression motif in an intervening region (IR) between the PR and CCT domains; however, this motif is not present in the IR of TOC1 [17].

Rhythmic gene expression and protein accumulation of PRR family members is crucial for proper clock function. *PRR*s are sequentially expressed from early daytime until around midnight, and each PRR protein works at a specific time. PRR9 functions during early daytime, PRR7 is active from early daytime until midnight, PRR5 works from noon until midnight, and TOC1 is expressed during nighttime [17]. The protein levels of the five PRRs in *Arabidopsis thaliana* peak sequentially throughout the day, starting with PRR9 3 to 4 h after dawn, followed by PRR7, PRR5, PRR3, and TOC1/PRR1 peaking 1 to 3 h after dusk [6, 7, 12, 15, 17]. PRR9, PRR7, PRR5, and TOC1 proteins are degraded during the night, so they mainly accumulate during the day when they repress the transcription of genes encoding other clock components such as *LHY* and *CCA1* [5, 6, 8, 19–21].

Research on different plants has contributed to our understanding of the function of PRRs. Dicotyledonous and monocotyledonous plants might share the evolutionarily conserved molecular mechanism underlying the circadian rhythm. Rice also has five members of the OsPRR family: OsPRR1, OsPRR73, OsPRR37, OsPRR95, and OsPRR59. Each of *OsPRRs* encodes a protein highly homologous to each one of the *AtPRR1/TOC1* quintet members: the *OsPRR1* gene is the ortholog of *AtPRR1*; *OsPRR95* and *OsPRR59* are classified into the same group, to which *APRR9*/*APRR5* belong; *OsPRR73*/*OsPRR37* considerably resemble either *APRR7* or *APRR3*, respectively [12]. There are five copies of the PRR genes in the genomes of *Sorghum bicolor*, *Vitis vinifera*, *Brassica rapa*, and *Carica papaya*, whereas seven *PRR* copies have been found in *Populus trichocarpa* [22–25]. Many *PRR* genes have been identified in a wide range of crop species. *Ppd-H1*, *Ppd-1*, *TaPRR73*, *BvBTC1*, *PRR37*, *DTH7*, *Ghd7.1*, *OsPRR37*, and *SbPRR37* encode PRR proteins in barley, wheat, beet, rice, and sorghum, respectively. Allelic variation at these genes confers natural diversity in flowering time [26–32]. Phylogenetic analysis indicates that there are three clades of *PRR* genes in land plants, including the *TOC1*, *PRR7/3*, and *PRR5/9* groups based on their similarity to their respective *Arabidopsis* proteins [33].

Although *PRR* genes have been investigated in various plants, few studies have explored these genes in maize, and information on the molecular characterization of the PRR family in maize (*Zea mays* L.) is minimal. Based on the assumption that other plants might share the molecular mechanisms underlying the circadian rhythm, we determined whether maize also has a set of PRRs and whether or not they are associated with the circadian rhythm. Systematical analyses including genome-wide identification, gene classification, chromosomal localization, gene structure analyses, phylogenetic analyses, and expression profiling were conducted in this study. The results might provide a basic understanding of the maize *ZmPRR* genes and facilitate future researches to further elucidate the potential function of *PRR* genes in maize development.

## 2. Materials and Methods

### 2.1. Plant Material, Photoperiod Treatment, and Tissue Collection

Maize inbred line CML288 plants, which is native to the tropical zone and highly sensitive to photoperiod, were planted in an artificial climate chamber at 28°C with photoperiod conditions of 12 h of light and 12 h of dark and an environmental humidity of 50%. To characterize the diurnal expression pattern of *ZmPRRs*, when the fifth leaves completely expanded, the plants were transferred to chambers at 28°C under SD conditions (9-h light, 15-h dark) and LD conditions (15-h light, 9-h dark), respectively. Ten days after SD or LD treatment, leaves were harvested in 3-h intervals during a 48-h period, starting at Zeitgeber time 0 (0 hr after the lights were turned on). Three biological replicates were collected at the same time. Then the samples were rapidly frozen in liquid nitrogen and immediately stored at -80°C until RNA extraction.

### 2.2. Identification of Putative PRR Genes in the Maize Genome

To identify all putative PRR proteins in the maize genome, a hidden Markov model profile and a BLAST search were performed. Each PRR gene contains the response regulator receiver domain of approximately 120 amino acids at its N-terminus and CCT motif of approximately 50 amino acids at the C-terminal end. Based on these criteria, HMMER search was performed by using the Pfam profile of response regulator receiver domain (PF00072) and CCT motif (PF06203) against the maize proteome sequence from the maize genome sequence project database to identify all putative PRR proteins in the maize genome. Simultaneously, the known sequences of PRR proteins from *Arabidopsis* and rice were also used as queries to search against the maize protein database with the BLASTP program. The AtPRR and OsPRR gene family information from *Arabidopsis* and *Oryza sativa* is listed in Table S1. *E*-value 1e^−10^ was used as the cut-off value in HMMER and BLAST searches. The candidate ZmPRR protein sequences were verified based on the presence of conserved response regulator receiver domain and CCT motif by searching against Pfam and SMART databases. Finally, redundant sequences were removed manually in terms of sequence alignment results. The basic physical and chemical parameters of each ZmPRR were calculated by the online ProtParam tool. Plant-mPLoc was used to predict the putative subcellular localization of maize PRR proteins [34].

### 2.3. Chromosomal Localization, Exon-Intron Structure Analysis, and Regulatory Cis-Element Analysis

All *ZmPRR* genes were mapped on chromosomes by identifying their chromosomal positions in the MaizeGDB database. The DNA and cDNA sequences corresponding to each predicted gene from the maize genome and annotation database were downloaded. The exon-intron structures of all genes were obtained using the online Gene Structure Display Server with coding and genomic sequences [35]. The cis-acting elements were searched from the 1500-bp sequences upstream of the start codon (ATG) of the nine members of the *ZmPRR* gene family by using the PlantCARE software [36].

### 2.4. Sequence Alignments, Phylogenetic Construction, and Motif Analysis

Multiple sequence alignments were performed on the ZmPRR family protein sequences using ClustalX2 with default parameters [37]. A phylogenetic tree was constructed with the aligned PRR protein sequences using MEGA7.0 by employing the neighbor-joining method with the pairwise deletion option and Poisson correction. Bootstrap analysis was performed using 1000 replicates on each node [38]. The MEME program was used to identify conserved motifs, with the following parameters: number of repetitions: any; the maximum number of motifs: 15; and the optimum motif widths: 6-200 amino acid residues [39]. Visualization of the motifs in proteins was created by TBtools [40].

### 2.5. Gene Duplications, Synteny Analysis, and Calculation of Ka/Ks Values and the Duplication Event Dating

MCScanX was applied to identify and analyze ZmPRR duplication and collinearity relationship among maize, rice, and sorghum, and the graph of synteny relationship was displayed by TBtools [40, 41]. The ratio of nonsynonymous to synonymous nucleotide substitutions (Ka/Ks) was calculated to evaluate the selection mode. The Ks values were further used to estimate the divergence time (*T*) of each duplicated ZmPRR gene pair, based on a rate of *λ* substitutions per synonymous site per year. Divergence times (*T*) were estimated with a formula *T* = Ks/2*λ* × 10^−6^ Mya (*λ* was kept as 6.5 × 10^−9^ as reported for grasses and used in earlier studies) [42].

### 2.6. Expression Pattern Analysis of the *ZmPRR* Genes

To elucidate the spatial and temporal gene expression patterns of *ZmPRR* genes, comprehensive expression analysis was accomplished based on publicly available genome-wide transcription data of maize different tissues at different developmental stages in the SRA (Sequence Read Archive) database at NCBI (National Center for Biotechnology Information) under the accession numbers PRJNA171684 and SRP010680, released by Stelpflug et al. [43]. FPKM (fragments per kilobase of exon per million fragments mapped) values from averaged biological replicates were used to measure the expression levels of genes. The average FPKM was then log2 transformed and used to generate a heat map using TBtools [40].

To carry on the diurnal expression profile analysis of *ZmPRR* genes, the relative expression levels of *ZmPRRs* during 48 h in inbred line CML288 plants under LD and SD conditions were detected by real-time quantitative PCR (qRT-PCR). Total RNA from leaves was extracted using TRIzol Reagent (Invitrogen, Carlsbad, CA, USA) according to the manufacturer's instruction. Each RNA sample was treated with DNase I to eliminate any residual genomic DNA. Then the RNAs were reverse transcribed into the first-strand cDNA by the RevertAid Premium Reverse Transcriptase (Thermo Scientific™ EP0733). Quantitative real-time PCR was performed on a LightCycler480 Software Setup (Roche) using SG Fast qPCR Master Mix (2X) (BBI) according to the manufacturer's instructions. Reaction volumes were 20 *μ*l consisting of 10 *μ*l SYBR Green qPCR Master Mix (2X), 0.4 *μ*l 10 *μ*M forward primer, 0.4 *μ*l 10 *μ*M reverse primer, 7.2 *μ*l ddH20, and 2 *μ*l cDNA template. Each reaction was performed in triplicate using the following conditions: 95°C for 3 min, followed by 45 cycles of denaturation at 95°C for 7 s, 57°C annealing for 10 s, and extending at 72°C for 15 s, followed by a melting-curve analysis to check the amplification specificity. The constitutive gene ubiquitin was used as endogenous control to normalize expression in maize tissues. Relative gene expression levels were calculated relative to ubiquitin using the 2^−*ΔΔ*Ct^ method. Gene-specific primers for qRT-PCR are presented in Supplementary Table S4.

## 3. Results

### 3.1. Genome-Wide Identification and Chromosomal Localization of *PRR* Genes in Maize

For the identification of all putative PRR proteins in the maize genome, HMMER search using the Pfam profile PF00072 (response regulator receiver domain) and PF06203 (CCT motif) as a query and BLASTP program using the known sequences of PRR proteins from *Arabidopsis* and rice as a query were performed by against the maize proteome sequence (B73 RefGen_v4) from the maize genome sequence project database. The basic information for *PRR* genes in *Arabidopsis* and rice is provided in Table S1. Only candidates that contained a PR domain at the N-terminus and a CCT motif at the C-terminal end were regarded as actual maize PRR proteins (ZmPRRs). As a result, nine candidate proteins were identified as members of the PRR gene family in maize (Table 1). To further verify the reliability of these candidate sequences, we performed the SMART analysis of the nine putative ZmPRR protein sequences, which detected the response regulator receiver domain and CCT motif sequences in all of these candidate sequences. For each of the PRRs identified in maize, a name was given according to the evolutionary relationship with PRRs in *Arabidopsis* and rice (see the section of phylogenetic analysis). We used the ProtParam tool from ExPASy to analyze the protein size, molecular weight, and theoretical isoelectric point (pI) of these ZmPRR proteins. These *ZmPRR* genes encode polypeptides ranging from 517 (*ZmPRR1-2*) to 766 (*ZmPRR73*) amino acid residues, with a transcript length from 2132 (*ZmPRR1-1*) to 3538 (*ZmPRR73*). The molecular weights of the identified ZmPRR proteins ranged from 57.56 kDa (ZmPRR1-2) to 83.76 kDa (ZmPRR73), and the theoretical isoelectric point varied from 6.12 (ZmPRR95-1) to 9.67 (ZmPRR37-1). The prediction of the subcellular localization of maize PRR proteins revealed that all these ZmPRRs were localized in the nucleus.

**Figure 1:**
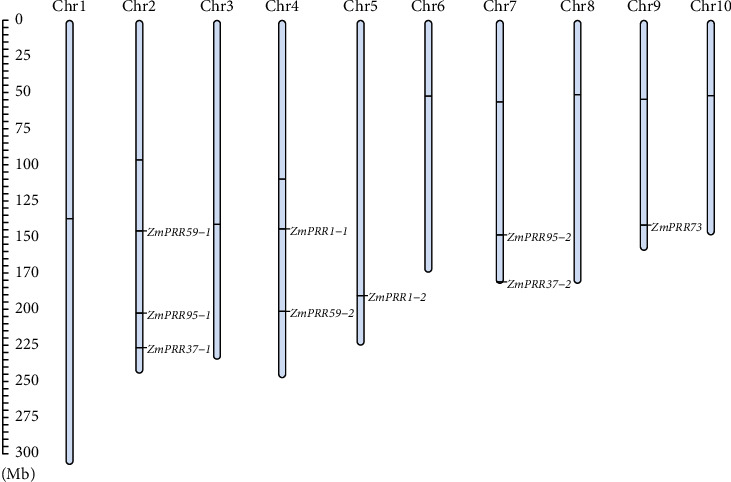
Chromosome distribution of *PRR* family genes in maize chromosomes. A total of 9 *ZmPRR* genes were mapped on the ten maize chromosomes with an uneven distribution. The chromosome numbers are indicated at the top of each bar. The gene names on the right side of each chromosome correspond to the approximate locations of each *ZmPRR* genes. The scale on the left is in megabases.

To gain insights into the evolution of the nine *ZmPRR* genes, we analyzed their genomic distribution. As shown in Figure 1, the nine putative *ZmPRR* genes were found on chromosomes 2, 4, 5, 7, and 9. Chromosome 2 contained three *ZmPRR* members (*ZmPRR59-1*, *ZmPRR95-1*, and *ZmPRR37-1*), chromosomes 4 and 7 contained two ZmPRR members, and chromosomes 5 and 9 only comprised one *ZmPRR* member.

### 3.2. Phylogenetic Analysis

**Figure 2:**
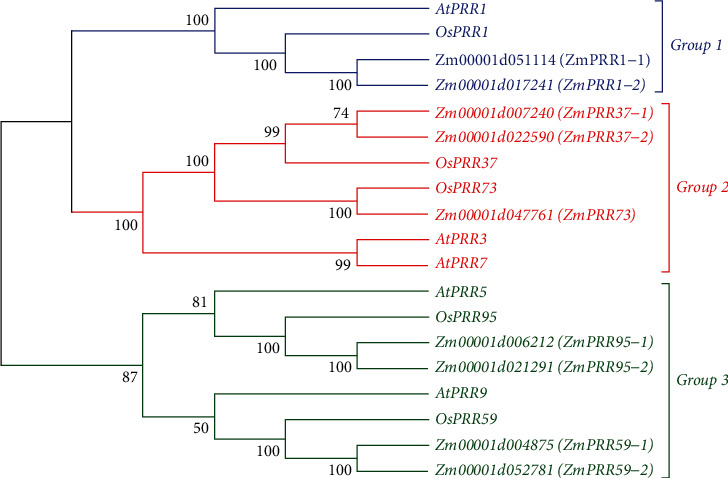
The phylogenetic tree of PRR proteins in maize, *Arabidopsis*, and rice.

To further study the evolutionary relationship between *ZmPRR* genes and *PRR* genes from other plants, a phylogenetic tree was constructed using the neighbor-joining algorithm with PRR amino acid sequences from *Arabidopsis*, rice, and maize by MEGA7. 19 PRR homologs, including 5 *Arabidopsis* PRRs, five rice PRRs, and 9 ZmPRRs were used to construct the phylogenetic tree (Figure 2). According to the generated unrooted phylogenetic tree, the PRR proteins were clustered into three distinct groups, the same as the classification of *OsPRR* genes (Murakami et al. 2003). *Zm00001d017241* and *Zm00001d051114* were classified into group 1, to which *AtPRR1* and *OsPRR1* belong, so they were designated as *ZmPRR1-1* and *ZmPRR1-2*, respectively. *Zm00001d007240*, *Zm00001d022590*, and *Zm00001d047761*were classified into group 2, to which *AtPRR3*, *AtPRR7*, *OsPRR37*, and *OsPRR73* belong. Group 2 was further subdivided into three subgroups. *Zm00001d007240* and *Zm00001d022590* were classed in the same subgroup with *OsPRR37*, so they were designated as *ZmPRR37-1* and *ZmPRR37-2*. *Zm00001d047761* was classed into the same subgroup with *OsPRR73*, so it was designated as *ZmPRR73*. Similarly, *Zm00001d004875*, *Zm00001d052781*, *Zm00001d006212*, and *Zm00001d021291* were classified into group 3, to which *AtPRR5*, *AtPRR9*, *OsPRR59*, and *OsPRR95* belong. *Zm00001d004875* and *Zm00001d052781* were classed into the same subgroup with *OsPRR59*, so they were designated as *ZmPRR59-1* and *ZmPRR59-2*, respectively. *Zm00001d006212* and *Zm00001d021291* were classed into the same subgroup with *OsPRR95*, so they were designated as *ZmPRR95-1* and *ZmPRR95-2*, respectively. In short, we here demonstrated that maize has nine *ZmPRR* genes (*ZmPRR1-1*, *ZmPRR1-2*, *ZmPRR37-1*, *ZmPRR37-2*, *ZmPRR73*, *ZmPRR59-1*, *ZmPRR59-2*, *ZmPRR95-1*, and *ZmPRR95-2*). Except for *ZmPRR73*, two of these genes were highly homologous to each of the *AtPRR* or *OsPRR* quintet members.

### 3.3. Intraspecies and Interspecies Synteny Analysis

To illustrate the evolutionary relationship of the *PRR* family, intraspecies and interspecies synteny analysis were conducted to identify duplicated gene pairs within maize genome and orthologous *PRR* genes pairs among maize, rice, and sorghum, respectively. Firstly, the whole genome synteny analysis was conducted to elucidate the origin of the *PRR* gene family in maize. Among the 9 *ZmPRR* genes, four duplicated pairs were detected, and most maize *PRR* genes were involved in gene duplication events except *ZmPRR59-1*. As expected, most maize *PRR* genes match the other *PRR* gene in the same group. The gene duplication events were analyzed to reveal the expansion mechanism of the maize *PRR* gene family. In this study, all of the duplicated pairs resulted from whole-genome duplication or segmental duplication, which suggested that whole-genome duplication or segmental duplication might be the main causes of *PRR* gene family expansion in maize.

To further explore the selection pressures on different duplicated *PRR* genes, the Ka and Ks substitution rates and the Ka/Ks values for each duplicated *ZmPRR* gene pair were calculated, respectively (Table 2). Ka/Ks ratio indicates what selection has been placed on this gene. It is commonly accepted that Ka/Ks = 1 indicated neutral selection, while Ka/Ks >1 means adaptive evolution with positive selection, and Ka/Ks < 1 means negative or purifying selection which means evolutionary pressure would eliminate deleterious mutations in the species to conserve the ancestral state. In our research, all the Ka/Ks values for *ZmPRR* duplicated gene pairs were < 1 indicating that the maize *PRR* gene family is highly conserved during evolution and are deduced to be under purifying selection which would eliminate deleterious mutations in the species. According to a substitution rate of 6.5 × 10^−9^ substitutions per synonymous site per year, as previously proposed for maize [42], the divergence time of 5 duplicated *ZmPRR* gene pairs was estimated to have occurred between 10.48 Mya (million years ago) and 163.93 Mya.

**Figure 3:**
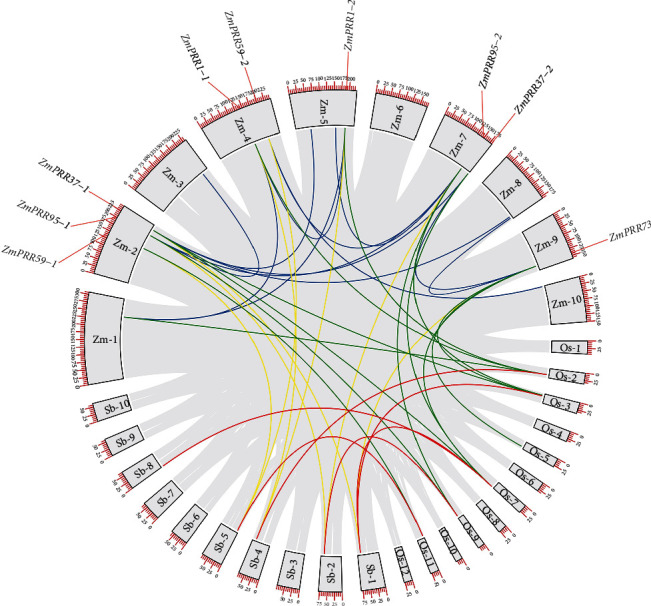
Synteny relationship of *PRR* regions across *Zea mays*, *Oryza sativa*, and *Sorghum bicolor*. The *Zea mays*, *Oryza sativa*, and *Sorghum bicolor* chromosomes are labeled as *Zm*, *Os*, and *Sb*, respectively. The *ZmPRRs* involved in duplications are mapped to their respective locations of the maize genome in the circular diagram. Numbers along each chromosome box indicate sequence lengths in megabases. Synteny blocks between maize and related grasses were detected and connected by lines in different colors. Blue lines represent the syntenic relationships between *ZmPRRs* and *ZmPRRs* regions. Green lines represent the syntenic relationships between *OsPRRs* and *ZmPRRs*. Yellow lines represent the syntenic relationships between *SbPRRs* and *ZmPRRs*. Red lines represent the syntenic relationships between *SbPRRs* and *OsPRRs*. Gray lines represent all syntenic regions in the whole genomes.

To further illustrate the evolutionary relationship of the *PRR* gene family, we applied comparative analysis to identify orthologous *PRR* genes pairs among maize, rice, and sorghum, respectively. As shown in Figure 3 and Table S2, we finally identified 10 orthologous gene pairs between maize and rice, whereas 8 orthologous gene pairs between maize and sorghum. More details about these orthologous gene pairs were shown in Table S2. As expected, the two *ZmPRR* genes in the same clade matched the same *PRR* genes in sorghum or rice, except that *ZmPRR59-2* did not match any orthologous gene in rice and *ZmPRR37-2* did not match any orthologous gene in sorghum.

### 3.4. Gene Structure Analysis and Identification of Conserved Motif

**Figure 4:**
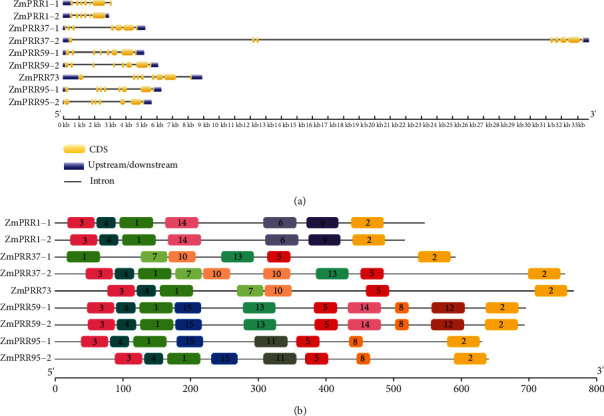
Distribution of exon-introns and conserved motifs of PRR genes in maize. (a) Phylogenetic relationship and distribution of exon/introns of PRRs in maize; (b) conserved motif predicated in ZmPRR proteins; the ZmPRR proteins are listed on the left. The solid black line represents the corresponding ZmPRR protein and its length. The different-colored boxes represent different motifs and positions in each ZmPRR protein sequence.

To study the structural diversity of *ZmPRR* genes, the exon/intron organization of individual *ZmPRR* genes was analyzed by comparing cDNA sequences with the corresponding genomic DNA sequence. The detailed gene structures are schematically shown in Figure 4(a). The number of introns ranges from 6 (*ZmPRR1-1*, *ZmPRR1-2*, *ZmPRR37-1*, and *ZmPRR37-2*) to 11 (*ZmPRR73*) in *ZmPRR* genes. Most *ZmPRRs* contained six or seven introns. Gene structure analysis indicated that these *ZmPRRs* are highly homologous to *AtPRRs* and *OsPRRs* not only in their amino acid sequences, but also in their structural designs. Two extraordinary long introns of 11562 and 18729 bp were found in *ZmPRR37-2*, which was much larger than that in other *ZmPRR* genes, which were consistent with the gene structure of *OsPRR37* and *OsPRR73* in rice. The amino acid sequence of ZmPRR1 is notably similar to that of AtPRR1 and OsPRR1. The *ZmPRR1-1* and *ZmPRR1-1* genes are unique in the sense that the coding sequence of their CCT motif is not separated by an intron, whereas other *ZmPRR* genes contain an extra intron which was consistent with *AtPRR1* and *OsPRR1* [12].

Conserved motif and gene structure analysis also verified the classification of the phylogenetic analysis. MEME4.12.0 was used to conduct conserved motif analysis, and 15 motifs were detected in ZmPRR proteins (Figure 4(b) and Table S3). The CCT motif (motif 2), motif 5, and the REC domain, composed of motif 3, motif1, and motif 4, were the most conserved motifs found in almost all nine ZmPRR members. It is worth noting that motif 3 and motif 4 were absent in ZmPRR37-1, whereas motif 5 was absent in ZmPRR1-1 and ZmPRR1-2. The genes in the same group usually share group specific conserved motifs. For instance, motif 6 and motif 9 were only found in ZmPRR1-1 and ZmPRR1-2, whereas motif 7 and motif 10 were only found in ZmPRR73, ZmPRR37-1, and ZmPRR1-2, and motif 8 and motif 15 were only found in ZmPRR59-1/-2 and ZmPRR95-1/-2. In general, the structure of ZmPRR proteins was conserved throughout the ZmPRR gene family.

To identify putative regulatory cis-acting elements enriched in maize PRR genes, the promoter sequences in 3 kb of genomic DNA upstream of the start codon (ATG) were extracted and searched against the PlantCARE database. Consistent with the fact that PRR is highly regulated by light, 26 putative cis-elements related to light response were detected in *ZmPRRs*. Sp1 and G-box were the most frequently found cis-elements in all the nine *ZmPRR* members, followed by circadian found in 5 ZmPRRs, and ACE and AE-box found in 4 ZmPRRs (Table 3). *ZmPRR73* had the highest number of light-responsive cis-elements, followed by *ZmPRR37-1*.

Thirteen putative cis-elements known to be regulated by hormones in some plant genes were identified in *ZmPRR* members: 3 cis-acting elements involved in abscisic acid responsiveness (ABRE, CE1, and CE3), 3 cis-acting elements participating in gibberellin-responsiveness (TATC-box, P-box, and GARE-motif), 3 cis-acting regulatory elements involved in auxin responsiveness (TGA-box, TGA-element, and AuxRR-core), 2 cis-acting regulatory elements associated with MeJA-responsiveness (CGTCA-motif and TGACG-motif), a cis-acting component associated with ethylene-responsive (ERE), and one cis-acting element involved in salicylic acid responsiveness (TCA-element) (Table 3). Among them, ABRE, CGTCA-motif, and TGACG-motif were the most frequently found cis-elements in all the nine *ZmPRR* members.

In addition, putative cis-elements associated with biotic and/or abiotic stress adaptive elements, such as anaerobic induction (ARE and GC-motif), cold and dehydration-responsiveness (C-repeat/DRE), heat stress responsiveness (HSE), low-temperature responsiveness (LTR), drought-inducibility (MBS), and defense and stress responsiveness (TC-rich repeats), were detected in a series of members.

### 3.5. The Expression Patterns of *ZmPRR* Genes in Different Tissue and Developmental Stages

**Figure 5:**
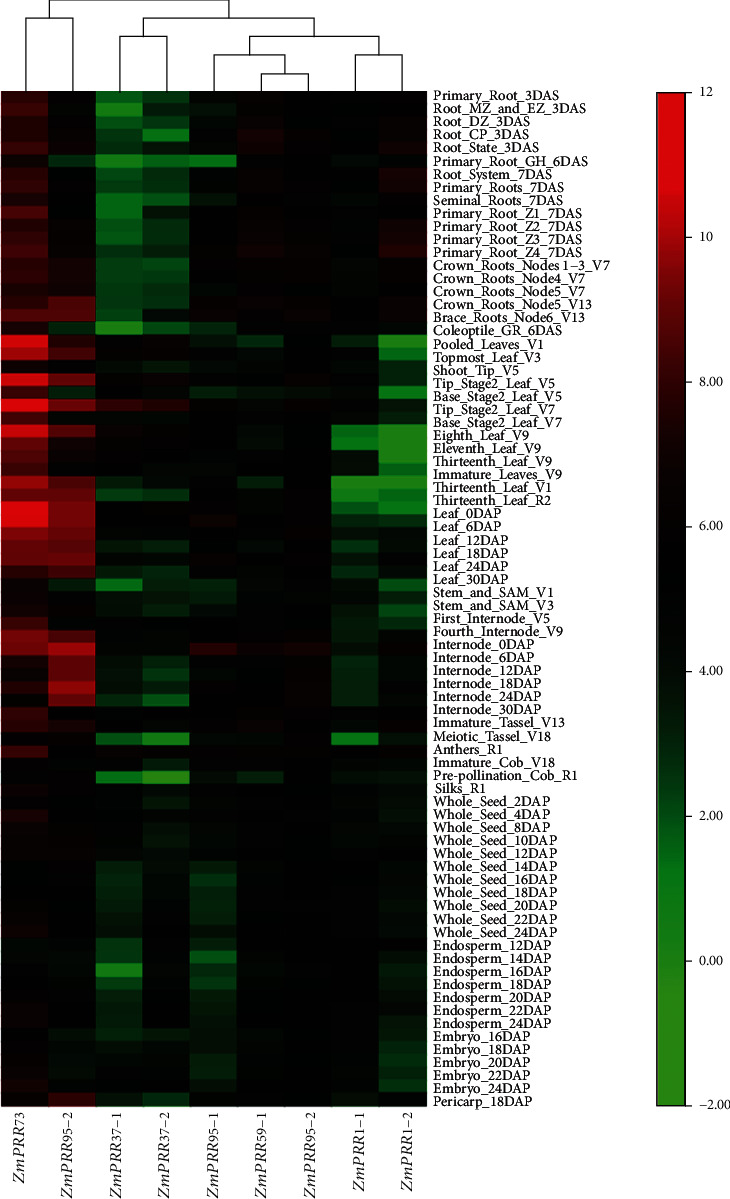
Expression profiles of *ZmPRR* genes across different tissues. The color scales for fold-change values are shown at the right. The expression values mapped to a color gradient from low (green) to high expression (red). The expression data were gene-wise normalized and hierarchically clustered.

To further investigate the tissue-specific expression patterns of *ZmPRR* genes, comprehensive expression analysis was accomplished based on publicly available genome-wide transcription data of maize tissues at different developmental stages in the SRA (Sequence Read Archive) database at NCBI (National Center for Biotechnology Information) under the accession numbers PRJNA171684 and SRP010680, released by Stelpflug et al. [43]. The tissue-specific expression patterns revealed the potential roles of *ZmPRRs* at maize special developmental stages. It can be seen from the heat map that nine *ZmPRR* genes express in all tissues and have distinct expression levels in different tissues at different developmental stages, as shown in Figure 5. Among the nine *ZmPRRs*, *ZmPRR73* showed the highest expression in almost all tested tissues, followed by *ZmPRR95-2*. All the *ZmPRRs* show relatively high expression in reproductive organs such as Immature_Tassel_V13, Meiotic_Tassel_V18, and Immature_Cob_V18. *ZmPRR37-1* and *ZmPRR37-2* exhibited relatively low expression levels in the root and seed at different developmental stages and relatively high expression levels in the leaves. The expressions of the remaining *ZmPRRs* were far lower. *ZmPRR1-2* exhibits a very low expression level in leaves at earlier developmental stages, whereas *ZmPRR1-1* exhibits far lower expression level in leaves after V9 developmental stages compared to other tissues. Specifically, *ZmPRR1-1* was expressed at high levels in endosperms of 12-16 days after pollination, which was remarkably more than any other *ZmPRRs*. *ZmPRR59-1*, *ZmPRR59-2*, and *ZmPRR95-2* show relatively high expression levels in all tissues except for *ZmPRR95-2* in seeds. Among all tested tissues, *ZmPRR73* exhibits the highest levels in leaves, and *ZmPRR95-2* exhibits the highest levels in internode. These results indicated the diverse functions among the members of the *ZmPRR* gene family in different stages of maize development.

### 3.6. The Expression Patterns of *ZmPRR* Genes under Diurnal Changes

**Figure 6:**
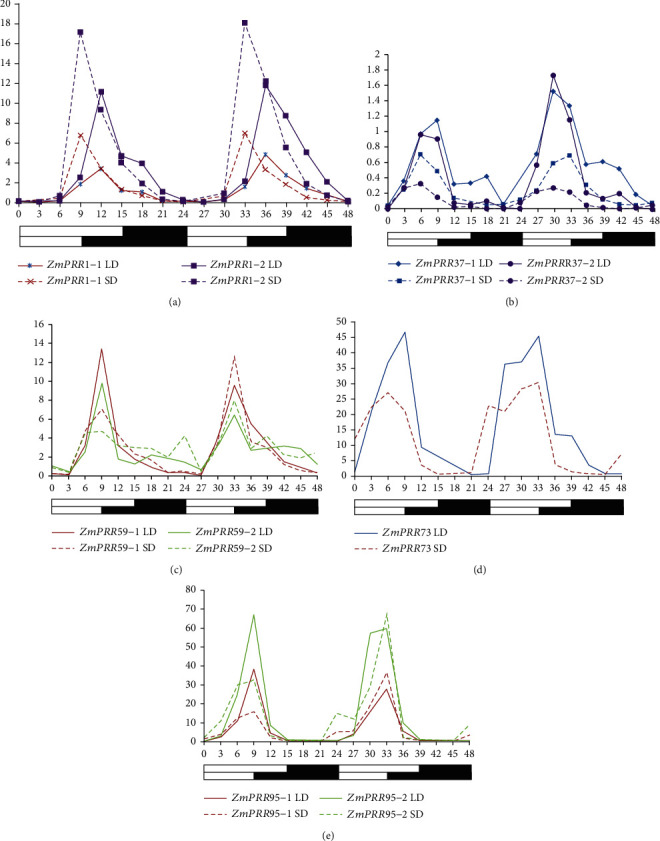
Expression pattern of *ZmPRRs* under diurnal changes. Expression levels of *ZmPRRs* in leaves of CML288 throughout a 48-h period of LD or SD treatment. The relative expression levels are normalized to *ZmUBQ*. The data are means ± SE of three biological replicates.

A feature shared by circadian genes is that their abundance is subject to diurnal oscillation. To characterize the diurnal expression pattern of *ZmPRRs*, CML288 plants were grown under LD and SD conditions, and the relative expression levels of *ZmPRRs* during 48 h were detected by qRT-PCR (Figure 6). qRT-PCR analysis revealed that the levels of *ZmPRRs* transcripts varied considerably and exhibited a diurnal rhythmic oscillation expression pattern in the given 24-h period under both SD and LD conditions. Each mRNA started accumulating after dawn sequentially at approximately 3-h intervals in the order of *ZmPRR73*, *ZmPRR37*, *ZmPRR95*, *ZmPRR59*, and *ZmPRR1*. The transcript levels *ZmPRR73* increased immediately from dawn and peaked 9 h after dawn and then gradually decreased under LD condition, while gradually increased at 3 h before dawn and peaked at 6-9 h after dawn before gradually declining in the afternoon SD conditions. The transcript levels *ZmPRR37-1* and *ZmPRR37-2* gradually increased after dawn in the morning and peaked at 6-9 h after dawn under both LD and SD conditions. The transcript levels *ZmPRR59-1*, *ZmPRR59-2*, *ZmPRR95-1*, and *ZmPRR95-2* gradually increased 3 h after dawn in the morning and peaked at 9 h after dawn regardless of the photoperiod conditions. The transcript levels *ZmPRR1-1* and *ZmPRR1-2* gradually increased at 6 h after dawn in the morning under both LD and SD conditions while reaching the peak three hours earlier under SD condition than LD condition. These results indicated that the level of transcription of *ZmPRRs* is subjected to a circadian rhythm and modulated by light and the circadian clock.

## 4. Discussion

### 4.1. Copy Numbers of *PRR* Family Genes in Maize

*PRR* genes are conserved among angiosperm evolutionary lineages. At least five *PRR* genes have been identified in angiosperm genomes. In eudicotyledonous plants, five copies of PRR genes have been identified in *A. thaliana*, *V. vinifera*, and *C. papaya*; seven copies have been found in *P. trichocarpa*, and eight copies have been identified in *B. rapa* [12, 22, 24, 44]. In monocotyledonous plants, there are five copies of *PRR* genes in the genomes of *O. sativa* and *S. bicolor*. In contrast to the five copy numbers in sorghum and rice, nine *ZmPRR* orthologs of the five *PRR* genes were identified and mapped to the chromosomes in maize in this study. This finding raises a complex question: why are the copy numbers of ZmPRRs different from *O. sativa* and *S. bicolor*?

Angiosperm genomes have undergone several whole-genome duplication (WGD) events. Maize, rice, and sorghum are members of the grass family (Poaceae), which share an ancient WGD dating to approximately 70 million years ago [45]. Maize and sorghum are members of the tribe Andropogoneae. These two species are estimated to have diverged approximately 12 million years ago, the same point in time as the initial diversification of Andropogoneae [46]. In addition to ancient WGD shared by all grasses, the maize genome has undergone a tetraploidy event approximately 5 to 12 million years ago after its divergence from sorghum, producing two functionally distinct maize subgenomes, maize 1, and maize 2 [47, 48]. The tetraploid history in maize resulted in the maize genome having a significantly higher number of orthologous genes than its close relative, *S. bicolor* and the core of the tribe Andropogoneae [45]. By contrast, sorghum and rice have experienced relatively few interchromosomal rearrangements since the ancient WGD shared by all grasses. Genes whose products participate in transcriptional regulatory networks are more likely to be retained as duplicate gene pairs after polyploidy events and subsequent gene deletion events because the imbalance associated with the loss of one member of transcription factors is likely to influence the regulatory network and downstream signals, which may decrease fitness [45, 48]. The regulatory network of the *Arabidopsis* clock system has maintained a degree of the organization throughout the dynamic changes of copy numbers and functions of clock-related genes [24]. Clock-related transcription factors, such as *CCA1/LHY/RVE* and *PRR* gene families, typically display preferential retention after WGD [4, 49, 50]. PRR genes in *P. trichocarpa* and *B. rapa* have expanded more than those in other plant species, and this expansion apparently resulted from polyploidy events. Some reports have shown that 43% of maize-sorghum syntenic genes are retained as homologous pairs in the maize genome. The frequency of genes encoding transcription factors is 4.3 times greater among the retained genes than the fractionated genes following WGD [51]. If the maize tetraploidy behaved as other known polyploidy in plants, the retained genes should be enriched in those clock-related transcription factors? Consistent with this hypothesis, nine *ZmPRR* orthologs were identified as members of the *PRR* gene family in maize in this study. Except for *PRR73*, two copies of each of the five *PRRs* were identified, which were significantly different from the copy number of the *PRR* gene family in other grass family members, such as rice and sorghum [24]. Circadian clocks may have become more intricate networks if additional genes have roles in circadian networks. However, the functions of these additional *ZmPRR* genes in the maize clock system, how these additional copies of *PRR* genes evolved in monocots, and how they are incorporated in the regulatory network of the clock system in the evolutionary history of maize remain to be elucidated.

### 4.2. Divergence of the Three *PRR* Clades Occurred Independently within Monocot and Eudicot Lineages

Ancient *PRR* genes have clearly diverged into three clades (*PRR1/TOC1* clade, *PRR3* and *PRR7* clade, and *PRR5* and *PRR9* clade) in angiosperms before the speciation of monocots and eudicots [24]. The copy numbers of *PRR* genes independently increased in each lineage as a result of ancient chromosomal duplication events. In most plant species, one copy of the *PRR1/TOC1* gene was retained in the *PRR1/TOC1* clade, whereas at least two copies were found in the *PRR3* and *PRR7* clade and the *PRR5* and *PRR9* clade. Phylogenetic analysis showed that the *PRR1/TOC1* clade consisted of two different clusters, each exclusively consisting of dicotyledonous *PRR1* or monocotyledonous *PRR1* genes, indicating that divergence of *PRR1* occurred after the speciation of monocots and dicots [24]. Although the *PRR3* and *PRR7* clade and the *PRR5* and *PRR9* clade contained two copies of *PRR* genes in monocots and dicots, the gene duplication events of the *PRR3* and *PRR7* clade and the *PRR5* and *PRR9* clade occurred independently within monocot and eudicot lineages, respectively [24]. In eudicots, the gene duplication events between *PRR3* and *PRR7* and between *PRR5* and *PRR9* are derived from the *γ* polyploidy event that took place before the speciation of *Vitales* (*V. vinifera*) and *eurosid* species. In monocots, the ancestral *PRR37/PRR73* was duplicated into *PRR37* and *PRR73* in the *ρ* polyploidy event that occurred before the speciation of *O. sativa* and *S. bicolor*. The monocot *PRR59* and *PRR95* genes showed an earlier gene duplication that may have occurred in a common ancestor of monocots and eudicots.

In this study, two copies of *PRR1/TOC1* genes, *ZmPRR1-1* and *ZmPRR1-2*, in the *PRR1/TOC1* clade were identified to be orthologous to *PRR1* genes. In the *PRR3* and *PRR7* clade, three copies of *PRR3* or *PRR7* genes were identified. *ZmPRR37-1* and *ZmPRR37-2* were identified to be orthologous to the monocotyledonous *PRR37* gene, whereas only *ZmPRR73* was identified to be orthologous to the monocotyledonous *PRR73* gene. Gene duplication events producing *ZmPRR37* and *ZmPRR73* might have occurred in a common ancestor of maize and rice. In contrast, the gene duplication events producing *ZmPRR37-1* and *ZmPRR37-2* occurred independently after the tetraploidy event in maize approximately 5 to 12 million years ago.

In the *PRR5* and *PRR9* clade, four copies of *PRR5* or *PRR9* genes were identified in maize. *ZmPRR59-1* and *ZmPRR59-2* and *ZmPRR95-1* and *ZmPRR95-2* were identified to be orthologous to the monocotyledonous *PRR59* and *PRR95* genes, respectively. The gene duplication events producing *PRR95* and *PRR59* or *PRR5* and *PRR9* may have occurred in a common ancestor of monocots and dicots. In the evolution process of maize, the *ZmPRR95* gene was duplicated into *ZmPRR95-1* and *ZmPRR95-2*, whereas the *ZmPRR59* gene was duplicated into *PRR59-1* and *PRR59-2* after the tetraploidy event in maize.

## 5. Conclusions

In this study, nine *PRRs* were identified and classified into three subgroups in maize based on phylogenetic evolution relationship, conserved motifs, and introns/exons analysis. In addition, *ZmPRRs* were systematically analyzed, including identification of conserved motif, the gene structure analysis, promoter cis-acting element predication, chromosome localization distribution, estimation of Ka/Ks, duplications, and collinearity analysis. Moreover, whole-genome duplication (WGD) and dispersed duplication (DSD) might be highly contributed to the expansion of *ZmPRRs*. Finally, the diurnal expression pattern of *ZmPRRs* under LD and SD conditions results and genome-wide transcription data of maize tissues at different developmental stages showed that the expression of *ZmPRRs* has a noticeable rhythmic expression trend and tissue-specific expression. It is speculated that *ZmPRRs* had a significant role in photoperiod response in maize. Our results provide us a strong base for studying the function of *PRRs* in maize.

## Figures and Tables

**Table 1:** The detailed characteristics of *ZmPRR* genes identified in maize.

Gene name	Gene ID	Location	Ave. residue weight (g/mol)	Charge	Isoelectric point	Molecular weight (g/mol)	Number of residues	Exon number	Transcript length (bp)	Grand average of hydropathicity	Subcellular location
*ZmPRR1-1*	Zm00001d051114	4:143867613:1438707	111.473	2.5	6.859	60864.39	546	7	2132	-0.593	Nucleus
*ZmPRR1-2*	Zm00001d017241	5:190060998:1900639	111.333	2	6.7272	57559.37	517	7	2169	-0.6	Nucleus
*ZmPRR37-1*	Zm00001d007240	2:225519590:2255243	107.252	22.5	9.6686	63492.96	592	7	2381	-0.933	Nucleus
*ZmPRR37-2*	Zm00001d022590	7:180509884:1805436	108.969	9	7.4884	82162.67	754	9	2838	-0.859	Nucleus
*ZmPRR73*	Zm00001d047761	9:141194049:1412029	109.353	1	6.5579	83764.05	766	12	3538	-0.887	Nucleus
*ZmPRR59-1*	Zm00001d004875	2:145683678:1456888	108.792	17.5	8.2365	75610.19	695	8	2750	-0.734	Nucleus
*ZmPRR59-2*	Zm00001d052781	4:200737914:2007440	108.216	18	8.2039	75101.76	694	8	2649	-0.63	Nucleus
*ZmPRR95-1*	Zm00001d006212	2:201957643:2019639	112.081	-4	6.1166	70722.9	631	9	2348	-0.82	Nucleus
*ZmPRR95-2*	Zm00001d021291	7:148065548:1480712	110.775	-1.5	6.3568	71006.56	641	10	2407	-0.681	Nucleus

**Table 2:** Ka/Ks analysis and estimated divergence time for the duplicated *ZmPRR* paralogs.

Paralogous pairs	Ka	Ks	Ka/Ks	Purifying selection	Divergence time (Mya)	Duplicate type
*ZmPRR95-1/ZmPRR95-2*	0.0582	0.1715	0.3396	Yes	13.19	WGD or segmental
*ZmPRR37-1/ZmPRR37-2*	0.0386	0.1362	0.2835	Yes	10.48	WGD or segmental
*ZmPRR37-2/ZmPRR73*	0.3274	1.2088	0.2708	Yes	92.98	WGD or segmental
*ZmPRR1-1/ZmPRR1-2*	0.0564	0.2290	0.2463	Yes	17.61	WGD or segmental
*ZmPRR59-2/ZmPRR95-2*	0.7097	2.1311	0.3330	Yes	163.93	WGD or segmental

**Table 3:** Putative cis-elements enriched in the promoters of *ZmPRR* family genes.

Site name	Sequence	Gene	Function
CE1	TGCCACCGG	*ZmPRR95-1*	Cis-acting element associated to ABRE, involved in ABA responsiveness
ABRE	TACGTGTC	*ZmPRR1-1/1-2/37-1/37-2/59-1/59-2/73/95-1/95-2*	Cis-acting element involved in the abscisic acid responsiveness
CE3	GACGCGTGTC	*ZmPRR37-1/59-1*	Cis-acting element involved in ABA and VP1 responsiveness
TGA-element	AACGAC	*ZmPRR1-1/37-1/95-1/95-2*	Auxin-responsive element
TGA-box	TGACGTAA	*ZmPRR1-1/59-1/59-2/95-1/95-2*	Part of an auxin-responsive element
AuxRR-core	GGTCCAT	*ZmPRR37-1/73*	Cis-acting regulatory element involved in auxin responsiveness
TCA-element	GAGAAGAATA	*ZmPRR1-1/37-1/37-2/59-2*	Cis-acting element involved in salicylic acid responsiveness
CGTCA-motif	CGTCA	*ZmPRR1-1/1-2/37-1/37-2/59-1/59-2/73/95-1/95-2*	Cis-acting regulatory element involved in the MeJA-responsiveness
TGACG-motif	TGACG	*ZmPRR1-1/1-2/37-1/37-2/59-1/59-2/73/95-1/95-2*	Cis-acting regulatory element involved in the MeJA-responsiveness
ERE	ATTTCAAA	*ZmPRR37-1/59-2*	Ethylene-responsive element
GARE-motif	AAACAGA	*ZmPRR1-2/37-2/59-2/73*	Gibberellin-responsive element
P-box	CCTTTTG	*ZmPRR1-1/1-2/37-2/59-2/73*	Gibberellin-responsive element
TATC-box	TATCCCA	*ZmPRR95-2*	Cis-acting element involved in gibberellin-responsiveness
MSA-like	CCCAACGGT	*ZmPRR37-1*	Cis-acting element involved in cell cycle regulation
CAT-box	GCCACT	*ZmPRR1-2/59-1/95-1/95-2*	Cis-acting regulatory element related to meristem expression
CCGTCC-box	CCGTCC	*ZmPRR1-2/59-1/95-1*	Cis-acting regulatory element related to meristem specific activation
Skn-1_motif	GTCAT	*ZmPRR1-1/37-1/37-2/59-2/73/95-1/95-2*	Cis-acting regulatory element required for endosperm expression
GCN4_motif	CAAGCCA	*ZmPRR37-1/37-2*	Cis-regulatory element involved in endosperm expression
CCAAT-box	CAACGG	*ZmPRR37-1/59-1*	MYBHv1 binding site
TC-rich repeats	ATTTTCTCCA	*ZmPRR1-1/1-2/37-1/59-1/59-2/95-1/95-2*	Cis-acting element involved in defense and stress responsiveness
HSE	AAAAAATTTC	*ZmPRR1-1/1-2/37-2/59-1/59-2/73*	Cis-acting element involved in heat stress responsiveness
ARE	TGGTTT	*ZmPRR37-2/73/95-1/95-2*	Cis-acting regulatory element essential for the anaerobic induction
GC-motif	CCCCCG	*ZmPRR1-1/1-2/37-2/59-1/73*	Enhancer-like element involved in anoxic specific inducibility
MBS	CAACTG	*ZmPRR1-1/1-2/37-1/59-2/73/95-1*	MYB binding site involved in drought-inducibility
C-repeat/DRE	TGGCCGAC	*ZmPRR1-1/1-2*	Regulatory element involved in cold- and dehydration-responsiveness
LTR	CCGAAA	*ZmPRR1-2/37-2/59-1/95-1*	Cis-acting element involved in low-temperature responsiveness
ACE	ACGTGGA	*ZmPRR1-1/1-2/73/95-2*	Cis-acting element involved in light responsiveness
Circadian	CAANNNNATC	*ZmPRR1-2/37-2/59-1/59-2/95-2*	Cis-acting regulatory element involved in circadian control
C-box	CTGACGTCAG	*ZmPRR37-2*	Cis-acting regulatory element involved in light responsiveness
G-box	CACGTT	*ZmPRR1-1/1-2/37-1/37-2/59-1/59-2/73/95-1/95-2*	Cis-acting regulatory element involved in light responsiveness
as-2-box	GATAatGATG	*ZmPRR1-2*	Involved in shoot-specific expression and light responsiveness
4 cl-CMA2b	TCTCACCAACC	*ZmPRR1-1/37-2*	Light responsive element
Box I	TTTCAAA	*ZmPRR37-1/59-2*	Light responsive element
GT1-motif	GGTTAA	*ZmPRR1-2/37-2/95-2*	Light responsive element
MNF1	GTGCCC(A/T)(A/T)	*ZmPRR37-1/73*	Light responsive element
Sp1	CC(G/A)CCC	*ZmPRR1-1/1-2/37-1/37-2/59-1/59-2/73/95-1/95-2*	Light responsive element
Box II	TCCACGTGGC	*ZmPRR37-1/73/95-1*	Part of a light responsive element
CATT-motif	GCATTC	*ZmPRR1-1/95-2*	Part of a light responsive element
chs-CMA2a	GCAATTCC	*ZmPRR73/59-2*	Part of a light responsive element
chs-CMA2b	GAACCTACACAC	*ZmPRR37-1*	Part of a light responsive element
chs-Unit 1 m1	ACCTAACCCGC	*ZmPRR59-1*	Part of a light responsive element
GAG-motif	AGAGATG	*ZmPRR37-1/59-2*	Part of a light responsive element
GA-motif	ATAGATAA	*ZmPRR37-1/73*	Part of a light responsive element
GATA-motif	AAGGATAAGG	*ZmPRR1-2/73*	Part of a light responsive element
I-box	acGATAATC	*ZmPRR37-1/73/95-1*	Part of a light responsive element
LAMP-element	CCAAAACCA	*ZmPRR73*	Part of a light responsive element
L-box	TCTCACCAACC	*ZmPRR1-1/37-2*	Part of a light responsive element
TCCC-motif	TCTCCCT	*ZmPRR37-1/73/95-1*	Part of a light responsive element
TCT-motif	TCTTAC	*ZmPRR1-2*	Part of a light responsive element
MRE	AACCTAA	*ZmPRR59-2/95-2*	MYB binding site involved in light responsiveness
ATC-motif	TGCTATCCA	*ZmPRR37-1/59-1/95-1*	Part of a conserved DNA module involved in light responsiveness
AE-box	AGAAACAA	*ZmPRR37-2/59-1/73/95-1*	Part of a module for light response
O2-site	GATGACATGG	*ZmPRR1-1/59-1/59-2/95-1/95-2*	Cis-acting regulatory element involved in zein metabolism regulation
EIRE	TTCGACC	*ZmPRR95-1*	Elicitor-responsive element

## Data Availability

The data used to support the findings of this study are available from the corresponding author upon request.

## Supplementary Materials

Supplementary Materials Table S1: the basic information for PRR genes in *Arabidopsis* and rice. Table S2: list of PRR orthologous gene pairs identified in maize, rice, and sorghum. Table S3: motif sequence of PRR genes family in maize. Table S4: list of primers used in qRT-PCR analysis.
