# Supplementary material for: Genome-Wide Identification and Characterization of PRR Gene Family and their Diurnal Rhythmic Expression Profile in Maize
Source: Int J Genomics. 2022 May 16;2022:6941607. doi: 10.1155/2022/6941607 (PMC9126661; doi:10.1155/2022/6941607)
Supplement: Supplementary Materials — Table S1: the basic information for PRR genes in Arabidopsis and rice. Table S2: list of PRR orthologous gene pairs identified in maize, rice, and sorghum. Table S3: motif sequence of PRR genes family in maize. Table S4: list of primers used in qRT-PCR analysis. [file 6941607.f1.docx]

Table S1. The basic information for PRR genes in Arabidopsis and rice

| gene locus | gene name | length bp | length aa | in/ex | Chr | location |
| --- | --- | --- | --- | --- | --- | --- |
| AT5G61380 | TOC1/APRR1 | 2808 | 618 | 5/6 | 5 | 24674963 - 24678550 |
| AT5G60100 | APRR3 | 2169 | 495 | 10/11 | 5 | 24197971-24201405 |
| AT5G24470 | APRR5 | 1677 | 558 | 5/6 | 5 | 8356204-8358546 |
| AT5G02810 | APRR7 | 3096 | 727 | 8/9 | 5 | 637681-642030 |
| AT2G46790 | APRR9 | 1952 | 468 | 6/7 | 2 | 19232607-19235179 |
| Os02g0618200 | OsPRR1 | 2260 | 518 | 5/6 | 2 | 24569489-24572391 |
| Os07g0695100 | OsPRR37 | 3311 | 742 | 10/11 | 7 | 29616705-29629215 |
| Os03g0284100 | OsPRR73 | 3873 | 745 | 9/10 | 3 | 9759666-9768689 |
| Os11g0157600 | OsPRR59 | 2342 | 697 | 7/8 | 11 | 2789011-2793728 |
| Os09g0532400 | OsPRR95 | 2398 | 623 | 7/8 | 9 | 20885173-20889792 |

Table S2. List of PRR orthologous gene pairs identified in maize, rice and sorghum

|  | maize | | rice | | sorghum | |
| --- | --- | --- | --- | --- | --- | --- |
| gene name | gene Id | location | gene Id | location | gene Id | location |
| ZmPRR1-1 | Zm00001d051114 | 4:145828378-145831004 | Os02t0618200 | 2:24569489-24572391 | SORBI_3004G216700 | 4:56625893-56628817 |
| ZmPRR1-2 | Zm00001d017241 | 5:189096673-189098971 | Os02t0618200 | 2:24569489-24572391 | SORBI_3004G216700 | 4:56625893-56628817 |
| ZmPRR37-1 | Zm00001d007240 | 2:225493109-225497780 | Os03t0284100 | 3:9759666-9768689 | SORBI_3001G411400 | 1:69433370-69440372 |
| ZmPRR37-1 | Zm00001d007240 | 2:225493109-225497780 | Os07t0695100 | 7:29616705-29629215 |  |  |
| ZmPRR37-2 | Zm00001d022590 | 7:183947053-183980196 | Os07t0695100 | 7:29616705-29629215 |  |  |
| ZmPRR73 | Zm00001d047761 | 9:144682004-144689285 | Os07t0695100 | 7:29616705-29629215 | SORBI_3001G411400 | 1:69433370-69440372 |
| ZmPRR73 | Zm00001d047761 | 9:144682004-144689285 | Os03t0284100 | 3:9759666-9768689 |  |  |
| ZmPRR95-1 | Zm00001d006212 | 2:202136411-202142127 | Os09t0532400 | 9:20885173-20889792 | SORBI_3002G275100 | 2:65784647-65790025 |
| ZmPRR95-2 | Zm00001d021291 | 7:149276295-149281474 | Os09t0532400 | 9:20885173-20889792 | SORBI_3002G275100 | 2:65784647-65790025 |
| ZmPRR59-1 | Zm00001d004875 | 2:145014636-145019177 | Os11t0157600 | 11:2789002-2793728 | SORBI_3005G044400 | 5:4190706-4195626 |
| ZmPRR59-2 | Zm00001d052781 | 4:202574180-202579719 |  |  | SORBI_3005G044400 | 5:4190706-4195626 |
|  |  |  |  |  |  |  |

Table S3 Motif sequenec of PRR genes family in maize

| Motif ID | Start | Width | *p*-value | Gene Name | Motif Seq |
| --- | --- | --- | --- | --- | --- |
| motif1 | 124 | 50 | 1.21E-60 | ZmPRR37-2 | CKNIPVIMMSSSDAMSTVFKCLSKGAVDFLVKPIRKNELKNLWQHVWRQR |
| motif1 | 19 | 50 | 7.65E-60 | ZmPRR37-1 | FKNIPVIMMSSSDDMSTVFKCLSKGAVDFLVKPIRKNELKNLWQHVWRQR |
| motif1 | 167 | 50 | 8.98E-60 | ZmPRR95-2 | SKNIPVIMMSSHDSVSMVFKCMLKGAADFLVKPIRKNELRNLWQHVWRKQ |
| motif1 | 126 | 50 | 4.89E-59 | ZmPRR59-1 | CKNIPVIMMSSQDSIGTVLKCMQKGAVDFLVKPVRKNELGNLWQHVWRRH |
| motif1 | 156 | 50 | 7.62E-59 | ZmPRR73 | CKDIPVIMMSTNDSMSMVFKCLSKGAVDFLVKPLRKNELKNLWQHVWRRC |
| motif1 | 117 | 50 | 1.57E-58 | ZmPRR95-1 | SKNIPVIMMSSHDSVSMVFKCMLKGASDFLVKPLRKNELRNLWQHVWRKQ |
| motif1 | 127 | 50 | 1.57E-58 | ZmPRR59-2 | CKNIPVVMMSSQDSIGTVLKCMQKGAVDFLVKPVRKNELRNLWQHVWRRH |
| motif1 | 101 | 50 | 4.83E-49 | ZmPRR1-2 | LRHIPIIMMSNRDEVSVVVKCLRLGAAEYLVKPLRTNELLNLWTHVWRRR |
| motif1 | 97 | 50 | 4.83E-49 | ZmPRR1-1 | LRHIPIIMMSNRDEVSVVVKCLRLGAAEYLVKPLRTNELLNLWTHVWRRR |
| motif2 | 591 | 49 | 5.32E-58 | ZmPRR95-2 | QREAALNKFRLKRKDRCFEKKVRYQSRKLLAEQRPRVKGQFVRQDHSIQ |
| motif2 | 581 | 49 | 5.32E-58 | ZmPRR95-1 | QREAALNKFRLKRKDRCFEKKVRYQSRKLLAEQRPRVKGQFVRQDHSIQ |
| motif2 | 710 | 49 | 2.46E-54 | ZmPRR73 | QREAALNKFRLKRKDRNFGKKVRYQSRKRLAEQRPRVRGQFVRQSEQED |
| motif2 | 637 | 49 | 6.85E-54 | ZmPRR59-2 | RREAALMKFRMKRKDRCFEKKVRYHSRKKLAEQRPRVKGQFVSQKLNSA |
| motif2 | 638 | 49 | 2.37E-53 | ZmPRR59-1 | HREAALMKFRMKRKDRCFDKKVRYHSRKKLAEQRPRVKGQFVSQKLKAA |
| motif2 | 439 | 49 | 2.26E-50 | ZmPRR1-1 | RRAAALAKFRLKRKERCFDKKVRYVNRKKLAETRPRVRGQFVRQPTNTD |
| motif2 | 538 | 49 | 8.72E-50 | ZmPRR37-1 | PRQAQLKKYREKKKDRNFGKKVRYQSRKRLADQRPRVRGQFVKQAVQNQ |
| motif2 | 700 | 49 | 1.75E-48 | ZmPRR37-2 | PRQEQLKKYREKKKDRNFGKKVRYQSRKRLADQRPRVRGQFVKQAVQNQ |
| motif2 | 441 | 49 | 9.40E-47 | ZmPRR1-2 | RRAAALAKFRQKRKERCFDKKVRYVNRKKLAETRLRVRGQFVRHASNMD |
| motif3 | 38 | 49 | 2.19E-53 | ZmPRR95-1 | MLPRMPVRVLLAEGDDSTRHVISALLRKCGYRVAAASDGVKAWDILKEK |
| motif3 | 88 | 49 | 8.36E-52 | ZmPRR95-2 | MLPRMPVRVLLAEGDDSTRHVISALLRKCGYRVAAASDGVKAWDLLKEK |
| motif3 | 48 | 49 | 8.70E-51 | ZmPRR59-2 | ILPRRSLRVLLVEHDDSTRQVVTALLRKCGYRVAAVADGMKAWGVMRER |
| motif3 | 47 | 49 | 3.81E-50 | ZmPRR59-1 | ILPRRSLRVLLVEHDDSTRQIVTALLRKCGYRVAAVADGMKAWEVMRER |
| motif3 | 77 | 49 | 3.41E-46 | ZmPRR73 | FLPVKTLRVLLVENDDSTRQVVSALLRKCCYEVIPAENGLHAWRYLEDL |
| motif3 | 45 | 49 | 1.70E-42 | ZmPRR37-2 | FLLKETLNVLLVESDDSTRQVVSALLRCCMYQVISAENGQQAWAYLEDK |
| motif3 | 22 | 49 | 2.59E-36 | ZmPRR1-2 | FVDRSKVRILLCDGDASSSREVLRLLCNCSYHVTCAKSPRQVINILNYE |
| motif3 | 18 | 49 | 6.67E-36 | ZmPRR1-1 | FVDRSKVRILLCDGDATSSREVLRLLCNCSYHVTCAKSPRQVINILNYE |
| motif4 | 88 | 20 | 6.10E-31 | ZmPRR95-1 | FNIDLVLTEVELPLMSGFLLLSTIMEHDA |
| motif4 | 127 | 20 | 2.24E-29 | ZmPRR73 | NNIDLVLTEVFMPCLSGIGLLSKITSHKI |
| motif4 | 138 | 20 | 3.56E-29 | ZmPRR95-2 | FNVDLVLTEVELPLMSGFLLLSTIMEHDA |
| motif4 | 95 | 20 | 1.77E-27 | ZmPRR37-2 | NNIDLVLTEVFMPGVSGISLLSRIMSHNI |
| motif4 | 98 | 20 | 8.86E-27 | ZmPRR59-2 | YAFDLVLTEVAMPSLSGIQLLSRIVAADE |
| motif4 | 97 | 20 | 9.99E-27 | ZmPRR59-1 | YDFDLVLTEVAMPSLSGIQLLSRIAAAAE |
| motif4 | 72 | 20 | 5.13E-23 | ZmPRR1-2 | GEIDIILAEVDLPVSKCFKMLKYIARNKD |
| motif4 | 68 | 20 | 5.13E-23 | ZmPRR1-1 | GEIDIILAEVDLPVSKCFKMLKYIARNKD |
| motif4 | 159 | 20 | 1.01E-18 | ZmPRR1-1 | DNFELVLSEPSDANTNSTTLLSDETDDRP |
| motif4 | 163 | 20 | 1.43E-18 | ZmPRR1-2 | DNFELVLSEPSDANTNSATLLSDETDDRP |
| motif5 | 315 | 45 | 2.11E-43 | ZmPRR37-1 | VLRRSNLSAFTRYHTSAASNQGGTGLVGSCSPHDNSSEAVKTDST |
| motif5 | 453 | 45 | 1.12E-42 | ZmPRR37-2 | ALRRSNLSAFTRYHTSAASNQGGTGLVGSCSPHDNSSEAVKTDST |
| motif5 | 461 | 45 | 2.59E-37 | ZmPRR73 | ILKRSDQSAFTRYHTSVASNQGGARYGESSSPQDNSSEAMKTDST |
| motif5 | 371 | 45 | 6.45E-35 | ZmPRR95-2 | TLNHSTSSAFSLYNCRAVPTLGNAGDGQLCSTSETLVDVENKNGD |
| motif5 | 358 | 45 | 2.63E-34 | ZmPRR95-1 | TLNHSTSSAFYLYNCRTASSLGNAGDGQLCSTSETLVDVENRNGD |
| motif5 | 384 | 45 | 2.92E-34 | ZmPRR59-1 | IFNHSNSSAFSRYGNRRIESSGEIQFLPSRCIVGQEQHVHGKDPV |
| motif5 | 385 | 45 | 5.29E-32 | ZmPRR59-2 | IFNHSNSSAFSRYGNKRIEPSGEIQFFPSPCVIGQGHVNGKNPVF |
| motif6 | 229 | 50 | 1.67E-61 | ZmPRR37-2 | CKKQKYTNDDFKGKDLEIDGPGNLYMDHQSSPNERPIKAADHENNSKESM |
| motif6 | 178 | 50 | 1.46E-57 | ZmPRR37-1 | CKKPKNTNDDFKGKDLEIGGPENLYMGHQSSPNGRSIKAADHENNSKESM |
| motif6 | 318 | 50 | 1.68E-56 | ZmPRR37-2 | VPEGKGKNDDFKGKDLEIGGPRNLYMDHQSSPNERPIKAADHENNSKESM |
| motif7 | 127 | 40 | 4.26E-50 | ZmPRR37-1 | SWTKLAVEIDSPQATSLDQLADPPNSTCAQVIHSKSEICS |
| motif7 | 178 | 40 | 1.21E-48 | ZmPRR37-2 | SWTKLAVEIDSPQAMSLDQLADPANSTCAQVIHSKSEICS |
| motif7 | 269 | 40 | 8.99E-46 | ZmPRR73 | SWTKRAVEIDSPQPISPDQLVDPPDSTCAQVIHPRSEICS |
| motif8 | 322 | 49 | 5.66E-50 | ZmPRR1-1 | SSNMHMERSSEGHNDTSGTPPAYHFPFYYPGMVEHNMALSSAQDFQANI |
| motif8 | 325 | 49 | 7.26E-50 | ZmPRR1-2 | SSSMHMERSNEGHNDTSGTPPAYHFPFYYPGMVEHNMALSSVQNFQANI |
| motif8 | 376 | 49 | 1.17E-46 | ZmPRR1-1 | HTPPAMLPQYNVYPQCHGLPVIPSFQFNPSGMSTHSSHLPTQNVWSSAS |
| motif8 | 379 | 49 | 9.00E-45 | ZmPRR1-2 | HTPPAMLHQYNVFSQCHSLPMISPFQFNTSGMSMHSSHLPTQNVWSSAS |
| motif9 | 480 | 45 | 1.95E-61 | ZmPRR59-1 | HRKDSMSHPSYGFVPVPIPVGAMMPYHCGAILQPVYYPQGPLMHC |
| motif9 | 480 | 45 | 8.43E-60 | ZmPRR59-2 | HRKDSMSHPSYGFIPVPIPVGATMPYHYGAILQPVYYPQSPLMHC |
| motif10 | 426 | 50 | 6.71E-67 | ZmPRR95-1 | PVQCFTFDGQPFWNGTPVASPFYPQSAPPIWNSKTPTWQESTPQATSLPQ |
| motif10 | 437 | 50 | 3.72E-62 | ZmPRR95-2 | PVQGLTFDGQPFWNGTPVASLFYSQSTPPIWNSKTSMWQESTPQATSLPQ |
| motif11 | 375 | 27 | 3.60E-33 | ZmPRR37-2 | TVRAADLIGSMAKNMDTQQAARAAEDT |
| motif11 | 286 | 27 | 2.76E-32 | ZmPRR37-2 | TVRAADLIGSMAKNMDAQQAARAADTP |
| motif11 | 235 | 27 | 3.56E-31 | ZmPRR37-1 | TVRAADLIGSMAKNMDTPQAARAAEDT |
| motif11 | 390 | 27 | 6.76E-18 | ZmPRR73 | NAETADLISSIARNTEGQQAVQAVDAP |
| motif12 | 309 | 50 | 9.45E-64 | ZmPRR95-2 | DMELVHIMENQQKYNTQWEVDTMRTTSRGNDEKGSIPAHQLELSLRRTDY |
| motif12 | 296 | 50 | 1.29E-61 | ZmPRR95-1 | DMELVHIMDNQQKHDTQRDVDTMRTTSRRNDEKNSIPAHQLELSLRRTDY |
| motif13 | 324 | 50 | 1.45E-45 | ZmPRR59-2 | DRKCQSSVMENNAVTENNPGDKSKGAAIGHADSCPSEFMVTNLGKEHHLN |
| motif13 | 176 | 50 | 3.55E-45 | ZmPRR59-1 | AMNCQTNGSENNAASNHVSTNVANGSKTGENNDEESDAQSFGNKRETEIK |
| motif13 | 177 | 50 | 2.04E-44 | ZmPRR59-2 | AMNCQTNGSENNAASNHISANVANGSKTGENSDEESDAQSFGSKRDTEIH |
| motif13 | 323 | 50 | 1.69E-41 | ZmPRR59-1 | DRKCQSLVMENNAVKEKNPGEKSKSAVIGHADSYPSQFLETNLGKQQYRN |
| motif14 | 272 | 49 | 1.01E-60 | ZmPRR1-1 | LQRGGSRLDSLDNQGNCSSATDRSDTGTDVNIRSKEAFEMPAQYPMVWF |
| motif14 | 275 | 49 | 1.18E-60 | ZmPRR1-2 | LQRGDSRLDSLDNHGNCSSATDRSDTGADVNIRNKEAFEMPVQYPMVCF |
| motif15 | 538 | 31 | 1.31E-39 | ZmPRR37-2 | ERVMLPSAIKANGYTSTFHPVQQWMVPDNAT |
| motif15 | 393 | 31 | 3.35E-37 | ZmPRR37-1 | TKPMLPSAIKANGYTSAFHPVQQWMVPANAT |
| motif15 | 545 | 31 | 7.98E-24 | ZmPRR73 | ERVASPLAIKSTQHASAFHTIQNQTSPANLI |

Table S4 List of Primers used in qRT-PCR analysis

| gene name | sequence | temperature | length |
| --- | --- | --- | --- |
| ZmPRR1-1-F | GGAGACGGATGCTTGGTTT | 57.2 |  |
| ZmPRR1-1-R | TTGAGGTGCCTGTTTCTTGAT | 57.3 | 162bp |
| ZmPRR1-2-F | ACCAACGCCTGACGAAACA | 59.4 |  |
| ZmPRR1-2-R | AAATTGACCTCGCACCCTTAG | 58.8 | 157bp |
| ZmPRR37-1-F | GGCATTCAGACCCAGAAGTGT | 58.8 |  |
| ZmPRR37-1-R | CATTATCACTGTCATCCCTTGC | 57.2 | 118bp |
| ZmPRR37-2-F | GGAGATGGTGCCAACGATAC | 57.5 |  |
| ZmPRR37-2-R | TGGAAGCCGCAGATGTATG | 57.5 | 106bp |
| ZmPRR73-F | AGGGAGCAGTTGATTTCTTGG | 58.6 |  |
| ZmPRR73-R | CAACACTGAAGTCGTCATCGTC | 57.9 | 222bp |
| ZmPRR59-1-F | CCGTGAAGATTGTGGTAGTGG | 57.9 |  |
| ZmPRR59-1-R | GTTGTCCTGATTCCCGATGA | 57.7 | 110bp |
| ZmPRR59-2-F | CACGCCGTCCAACAAATC | 57.5 |  |
| ZmPRR59-2-R | CTTCGACTTATCACCAGGGTTAT | 57.4 | 125bp |
| ZmPRR95-1-F | GAACAGAAGACTGGCGTGACA | 58.3 |  |
| ZmPRR95-1-R | TTTGCTTACTTTCAGCCTCCA | 58 | 136bp |
| ZmPRR95-2-F | CATCAATGTGGCAAGAATCAAC | 58 |  |
| ZmPRR95-2-R | CCACTTTCACCAGTCATAGGAGA | 58.6 | 208bp |
| ZmUbiquitin-F | GTCTCCGTGGTGGTCAGTAAGT | 58.1 |  |
| ZmUbiquitin-R | GACACGAACAGCAGATACTTTGAC | 58.6 | 107bp |
